# Neonatal diffusion tensor brain imaging predicts later motor outcome in preterm neonates with white matter abnormalities

**DOI:** 10.1186/s13052-016-0309-9

**Published:** 2016-12-01

**Authors:** Do-yeon Kim, Hyun-Kyung Park, Nam-Su Kim, Se-Jin Hwang, Hyun Ju Lee

**Affiliations:** 1 Department of Pediatrics, Hanyang University College of Medicine, Seoul, South Korea; 2 Division of Neuroanatomy, Department of Anatomy and Histology, Hanyang University College of Medicine, Seoul, South Korea

**Keywords:** Neonates, Magnetic resonance imaging, Diffusion tensor imaging, Periventricular leukomalacia

## Abstract

**Background:**

White matter (WM) abnormalities associated with prematurity are one of the most important causes of neurological disability that involves spastic motor deficits in preterm newborns. This study aimed to evaluate regional microstructural changes in diffusion tensor imaging (DTI) associated with WM abnormalities.

**Methods:**

We prospectively studied extremely low birth weight (ELBW; <1000 g) preterm infants who were admitted to the Neonatal Intensive Care Unit of Hanyang University Hospital between February 2011 and February 2014. WM abnormalities were assessed with conventional magnetic resonance (MR) imaging and DTI near term-equivalent age before discharge. Region-of-interests (ROIs) measurements were performed to examine the regional distribution of fractional anisotropy (FA) values.

**Results:**

Thirty-two out of 72 ELBW infants underwent conventional MR imaging and DTI at term-equivalent age. Ten of these infants developed WM abnormalities associated with prematurity. Five of ten of those with WM abnormalities developed cerebral palsy (CP). DTI in the WM abnormalities with CP showed a significant reduction of mean FA in the genu of the corpus callosum (*p* = 0.022), the ipsilateral posterior limb of the internal capsule (*p* = 0.019), and the ipsilateral centrum semiovale (*p* = 0.012) compared to normal WM and WM abnormalities without CP. In infants having WM abnormalities with CP, early FA values in neonatal DTI revealed abnormalities of the WM regions prior to the manifestation of hemiparesis.

**Conclusions:**

DTI performed at term equivalent age shows different FA values in WM regions among infants with or without WM abnormalities associated with prematurity and/or CP. Low FA values of ROIs in DTI are related with later development of spastic CP in preterm infants with WM abnormalities.

**Electronic supplementary material:**

The online version of this article (doi:10.1186/s13052-016-0309-9) contains supplementary material, which is available to authorized users.

## Background

Despite the recent advances in both antenatal and neonatal intensive care, neurodevelopmental outcomes in those born prematurely have improved little over time. Many studies have reported that neurodevelopmental disorders observed in preterm infants comprise motor and cognitive impairment, language delays, behavioral disorders, and psychological problems [1–3].

White matter (WM) abnormalities associated with prematurity are the predominant cause of neurological disabilities in preterm infants. Periventricular foci of necrosis in preterm infants are caused by multifactorial insults including hypoxia-ischemia, infection/inflammation and coagulation disturbance at a particular timing of brain development [4]. Early prediction of motor and cognitive deficits is crucial to recognize patients with WM injury who will benefit from early developmental intervention programs, which offer the possibility of improving the neurological outcomes. Although magnetic resonance imaging (MRI) has provided insight into the underlying WM injury, compared to cranial sonography, structural MRI studies fail to quantitatively measure microstructural abnormalities and predict outcomes during the neonatal period [5].

The diffusion tensor imaging (DTI) of advanced MRI reflects changes in WM connection and myelination by the detection of water anisotropy according to the degree and direction of water molecule permeability in tissues. Fractional anisotropy (FA) is used to measure the directionality obtained in axon bundles as well as myelination. Increasing evidence has suggested that the low FA values in WM association areas are related to negative motor and cognitive functions in preterm infants [6].

Nevertheless, few studies have been conducted to evaluate the correlations between WM connectivity as revealed by DTI and motor neurodevelopment of extremely low birth weight (ELBW; <1000 g) infants with WM abnormalities [7, 8]. Therefore, this study aimed to determine the diffusion tensor characteristics of WM regions associated with motor outcome among preterm infants with or without WM abnormalities and/or cerebral palsy (CP).

## Methods

This study is part of a prospective research program on ELBW infants involving short- and long-term postnatal follow-up at the Hanyang Inclusive Clinic for Developmental Disorders in Hanyang University College of Medicine. The 72 ELBW infants (<1000 g) born and admitted to a level 3 Neonatal Intensive Care Unit at Seoul Hanyang University Hospital of South Korea between February 2011 and February 2014 were eligible for the study. The major exclusion criteria were congenital malformations or chromosomal anomalies. These infants were imaged during natural sleep without sedation using oral chloral hydrate.

### Clinical characteristics of study infants

Prenatal and neonatal data were prospectively recorded, including gestational age (GA), birth weight, delivery mode, sex, twin status, Apgar at 5 min, maternal chorioamnionitis, and prenatal steroid use for each infant. Chorioamnionitis was defined by the presence of histologic chorioamnionitis or umbilical cord vasculitis of grade 2 or greater, using the grading system suggested by Salafia et al. [9]. Neonatal outcomes included patent ductus arteriosus, bronchopulmonary dysplasia (BPD), culture-proven sepsis, necrotizing enterocolitis, retinopathy of prematurity, intraventricular hemorrhage (IVH) (grade ≥ III) according to the Papile classification [10], and CP. The diagnosis and severity of BPD were based on the need for supplementary oxygen at 28 days of age and at 36 weeks gestational age [11]. Intraventricular hemorrhage was defined according to Volpe [10], and necrotizing enterocolitis was defined according to Bell et al. [12]. CP was defined as a classification proposed by the Surveillance of CP in Europe (SCPE) collaborative group. Spastic CP was diagnosed if they had at least two of the following criteria: abnormal posture or movement, increased tone, or hyperreflexia [13]. The diagnosis of unilateral or bilateral spastic CP was made by the rehabilitation physician and, when necessary, confirmed by a neuropediatrician at the corrected age of 24 months at follow-up. All preterm infants who underwent a DTI exam were categorized into the “no WM abnormalities” group, the “WM abnormalities without CP” group, or the “WM abnormalities with CP” group to identify the differences in the clinical characteristics and FA values on DTI according to the regions of interest among these groups. Radiological evaluation was performed by an experienced pediatric neuroradiologist, Y.L., who was blinded to all clinical data. Brain abnormalities were assessed on structural MRI for presence/absence of WM abnormalities and details on co-existing types of lesions.

### MRI data analysis (term-equivalent)

Conventional magnetic resonance (MR) images and diffusion tensor images were obtained with a 3.0 T MRI scanner (Philips Real Time Compact Magnet 3.0-Tesla MRI system, Achieva 3.0-Tesla X-series) with a six-channel SENSE head coil operating. Conventional MR images included sagittal and axial T1 spin-echo sequences (400/25/2, TR/TE/signal intensity averages) and axial T2 fast spin-echo (4500/90/3). The Philips Research Imaging Development Environment (PRIDE) Diffusion Registration tool (version 0.4) was used to calculate FAs of the diffusion tensor data after processing of the DT-MRI images. Region-of-interests (ROIs) measurements were performed to examine the regional distribution of FA values in 2-dimensional space. We reconstructed fiber-tracking in 3-dimensional space using PRIDE Fiber Tracking tool (version 4.1) and set 3 ROIs for the motor tract at the ventral part of the pons, the internal capsule and at the centrum semiovale to evaluate fiber connectivity. We didn’t include a tractography to analyze quantitatively but performed tractography itself to assess the connectivity and disruption of depicted motor tract.

Diffusion tensor images were sing a single-shot spin-echo planar sequence with a SENSE factor of two and an echo-planar imaging factor of 67 (TR/TE, 13891/55 ms; matrix size, 112 × 112; field-of-view, 224 mm; 90 axial sections; 2.0-mm section thickness). Diffusivities were measured along 15 directions using an electrostatic gradient model (*b* = 800). Tracking was stopped when the FA in a pixel below 0.18 was reached to prevent streamlines from going into low anisotropy gray matter.

**Fig. 1:**
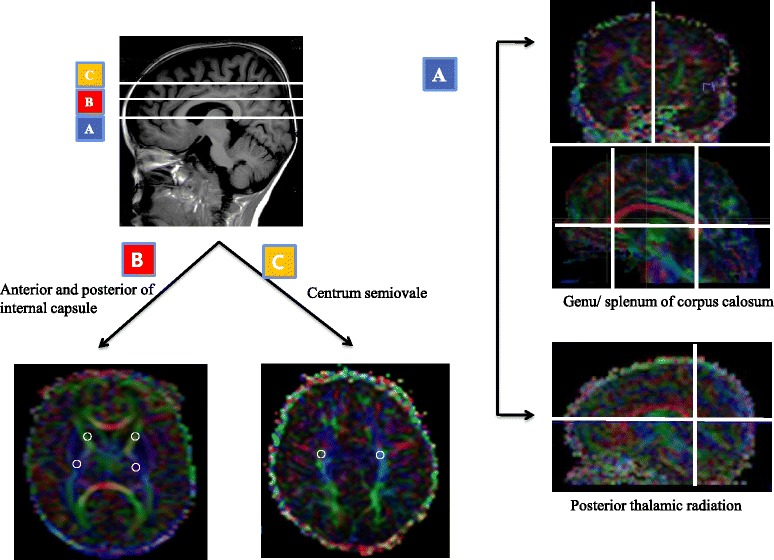
A region-of-interest placement of the white matter tract selected on a representative infant diffusion tensor imaging at near-term age

To ensure reliability, consensus on each region of interest placement and measurement was reached by two independent researchers. Tracking was initiated by manually placing a region of interest within anatomically similar regions of the corpus callosum (genu and splenum), anterior internal capsule, posterior internal capsule, posterior thalamic radiation, and centrum semiovale (Fig. 1).

### Neurodevelopmental assessment

Neurodevelopmental outcomes were assessed at a mean age of 18 ± 3.5 months (range: 15–23 months) with the Bayley Scale for Infant Development-III (BSID-III), which evaluates five distinct scales: cognitive; language, with receptive and expressive communication subtests; motor, with fine and gross motor subtests; socioemotional behavior; and adaptive behavior. The average BSID-III score in healthy infants and children is 100 ± 15.

### Statistical analysis

Comparisons between groups were carried out by one-way Analysis of Variance or Kruskal-Wallis tests for comparison of continuous variables. Categorical variables were analyzed by Pearson’s chi-square test or Fisher’s exact test (both two-sided), as appropriate. To account for multiple comparisons, Bonferroni’s correction was considered. All statistical analyses were carried out using SPSS 17.0 (SPSS Inc.). *P*-values < 0.05 were considered statistically significant. The study was approved by the Hanyang University Hospital Institutional Review Board, and written informed consent was obtained from the patients’ parents.

## Results

Seventy-two infants with ELBW were admitted during the study period, and 62 infants were included after parental consent was obtained. Eighteen infants were excluded due to instability during the MRI exam with poor results, and two infants were excluded due to insufficient data. Excluding deaths (*n* = 10) and refusals (*n* = 10), 32 patients who fulfilled the study criteria were enrolle. During the study period, 32 infants with available DTI data were evaluated for the regional distribution of FA values associated with WM injury with GAs ranging between 23 and 30 weeks and birth weights ranging between 760 and 1740 g. Thirty-two ELBW infants (19 males and 13 females) underwent conventional MRI and DTI at a mean post-menstrual age of 36.5 ± 1.9 weeks. Ten of these infants developed WM abnormalities (bilateral: 4, left side: 6). Five of the ten infants with WM abnormalities (bilateral: 2, left side: 3) developed spastic CP (bilateral: 2, unilateral: 3). The infants enrolled in the DTI analysis were classified into three groups; no WM abnormalities, WM abnormalities without CP, and WM abnormalities with CP. Table 1 shows the clinical characteristics and neonatal outcomes of the groups. The mean gestational age and birth weight were not significantly different among the no WM abnormalities, WM abnormalities without CP, and WM abnormalities with CP groups. The infants in the WM abnormalities with CP group showed a higher occurrence of any grade of IVH and of IVH grade ≥ III (*P* < 0.001).

**Table 1:** Clinical characteristic of study infants

	No WM^a^ (*N* = 22)	WM^a^ without CP (*N* = 5)	WM^a^ with CP (*N* = 5)	*P*-value
Gestational age (wk)	26.14 ± 2.44	27.40 ± 2.79	25.00 ± 1.22	0.292
Birth weight (g)	819 ± 133	770 ± 153	738 ± 146	0.441
Cesarean section, *n* (%)	17 (77.2)	5 (100)	5 (100)	0.335
Male gender, *n* (%)	15 (68.2)	2 (40)	2 (40)	0.322
Twin, *n* (%)	7 (31.8)	3 (60)	0 (0)	0.122
Apgar score at 5 min	4.27 ± 1.42	4.60 ± 0.55	5.00 ± 1.22	0.516
Chorioamnionitis, *n* (%)	13 (59.1)	4 (80)	3 (60)	0.678
Prenatal steroid use, *n* (%)	19 (86.4)	4 (80)	4 (80)	0.900
Hospital Days	95.25 ± 22.71	64.80 ± 38.36	70.75 ± 39.94	0.072
Days on ventilation	22.59 ± 15.56	11.60 ± 4.16	32.20 ± 20.12	0.121
PDA, *n* (%)	18 (81.8)	4 (80)	5 (100)	0.606
BPD ≥ moderate, *n* (%)	7 (31.8)	3 (60)	2 (40)	0.497
Sepsis, *n* (%)	10 (45.5)	1 (20)	3 (60)	0.426
NEC, *n* (%)	4 (18.2)	0 (0)	1 (20)	0.575
IP, *n* (%)	3 (13.6)	0 (0)	0 (0)	0.471
ROP ≥ grade 2, *n* (%)	13 (59.1)	3 (60)	1 (20)	0.137
IVH, *n* (%)	8 (36.4)	4 (80)	5 (100)	0.015
IVH, grade III/IV, *n* (%)	2 (9.1)	3 (60)	5 (100)	<0.001
Cerebral palsy, *n* (%)	0	0	5 (100)	<0.001

Data are presented as mean ± SD or number (%)

*Abbrevations*: *WM* ^*a*^ white matter abnormalities, *PDA* patent ductus arteriosus, *BPD* bronchopulmonary dysplasia, *NEC* necrotizing enterocolitis, *IP* intestinal perforation, *ROP* retinopathy of prematurity, *IVH* intraventricular hemorrhage, *PVL* periventricular leukomalacia, *CP* cerebral palsy

**Fig. 2:**
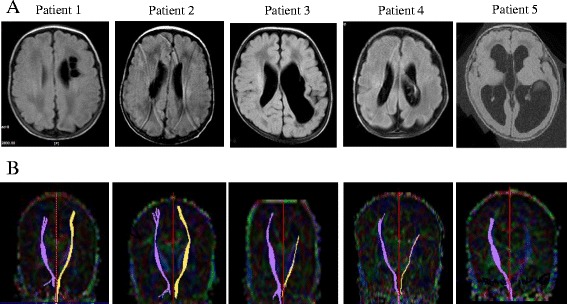
The representative axial images on T2 flair image (**a**) and tractography of motor fibers on DTI (**b**) are shown in preterm infants with white matter abnormalities and cerebral palsy

FA values of ROIs in the DTI showed that the genu, anterior/posterior limb of the internal capsule, bilateral posterior thalamic radiation, and centrum semiovale were attenuated in the WM abnormalities groups. In addition, DTI parameters in the WM abnormalities with CP showed a significant reduction of mean FA in the genu of the corpus callosum (*p* = 0.022), the ipsilateral posterior limb of the internal capsule (*p* = 0.019), and the ipsilateral centrum semiovale (*p* = 0.012) compared to those in no WM abnormalities group and WM abnormalities without CP group. Although there were no significant differences in the splenum of the corpus callosum between the study groups, the WM abnormalities with CP group had lower FA values compared to the WM abnormalities without CP group (Table 2). In infants with WM abnormalities and CP, patient 1–2 showed successful assessment of bilateral motor fiber tracts, whereas patient 3–4 displaced reduced or disrupted fiber tracts in the left side. Fiber tracts were not delineated in the left side in patient 5 (Fig. 2). The representative axial images on T2 flair image (A) and tractography of motor fibers on DTI (B) are shown in preterm infants with white matter abnormalities without cerebral palsy (Additional file 1).

**Table 2:** Fractional anisotropy values of study infants with diffusion tensor imaging

Region-of-interest	No WM^a^ (*N* = 22)	WM^a^ without CP (*N* = 5)	WM^a^ with CP (*N* = 5)	*P*-value
Genu of Corpus Callosum	0.32 ± 0.06	0.30 ± 0.01	0.24 ± 0.02	0.022
				0.020^*^
Splenum of Corpus Callosum	0.34 ± 0.06	0.33 ± 0.04	0.27 ± 0.04	0.068
Rt. Anterior Internal Capsule	0.31 ± 0.51	0.28 ± 0.07	0.28 ± 0.04	0.345
Lt. Anterior Internal Capsule	0.29 ± 0.03	0.26 ± 0.04	0.26 ± 0.03	0.131
Rt. Posterior Internal Capsule	0.36 ± 0.49	0.32 ± 0.47	0.31 ± 0.07	0.108
Lt. Posterior Internal Capsule	0.36 ± 0.04	0.35 ± 0.01	0.30 ± 0.06	0.019
				0.016^*^
Rt. Posterior Thalamic Radiation	0.29 ± 0.04	0.25 ± 0.03	0.26 ± 0.08	0.140
Lt. Posterior Thalamic Radiation	0.30 ± 0.04	0.26 ± 0.01	0.28 ± 0.05	0.144
Rt. Centrum Semiovale	0.34 ± 0.04	0.31 ± 0.04	0.31 ± 0.03	0.170
Lt. Centrum Semiovale	0.33 ± 0.05	0.30 ± 0.01	0.26 ± 0.02	0.012
				0.011^*^

Data are presented as mean ± SD or number (%)

*Abbrevations*: *WM* ^*a*^ white matter abnormalities, *PVL* periventricular leukomalacia, *CP* cerebral palsy, *Lt* left, *Rt* right

^*^ P comparing No PVL group and PVL with CP group in the Bonferroni’s correction for multiple comparisons

In infants having WM abnormalities with CP, DTI at discharge revealed abnormalities of FA values in WM regions prior to the manifestation of abnormal motor function and/or impaired cognition. Five children displayed spastic CP (bilateral: 2, unilateral: 3) and/or impaired cognition (four out of five children). The remaining five infants constituted the WM abnormalities without CP group. Four of these five children had normal development without delays of cognitive, motor and language functions as assessed with the BSID-III.

## Discussion

This study demonstrated that DTI performed at term equivalent age shows different FA values in WM regions among infants with or without WM abnormalities associated with prematurity and/or CP. The motor outcome of the patients with WM abnormalities associated with prematurity was associated with low FA values in the DTI parameters of the genu of the corpus callosum, the ipsilateral posterior limb of the internal capsule, and the centrum semiovale at discharge in extremely low-birth weight infants.

WM abnormalities associated with prematurity, which is the leading cause of CP, is estimated to occur in 10–15% of very low-birth weight (VLBW; <1500 g) infants and is attributed to the developing brain’s vulnerability to hypoxic ischemic events [14]. Follow-up studies reported that 20–40% of children born with VLBW had isolated cognitive deficiencies, even in cases without significant cerebral damage, resulting in impaired language skills, learning, executive functions, or social abilities [15–17]. Few studies have clearly shown that the extent of structural abnormalities, microstructural deviations, and global reductions in brain volumes, both at preterm and term ages, is directly related to the level of neuromotor and neurocognitive performance in childhood [18, 19].

Great progress has been made in the past few decades in the approach to microstructural development with a novel tract-based analysis of DTI data in infants. DTI has good sensitivity and specificity to assess quantitative changes in the various brain microstructures during the developmental stage [20]. Conventional MRI has been limited in the quantitative evaluation of specific WM tracts in the premature brain. While conventional MRI is able to visualize only macroscopic characterization of WM after the myelination, DTI is sensitive to the maturational changes in premyelinating WM prior to the onset of myelination [21, 22]. Although the DTI image analysis of various brain structures in the early developmental phases is challenging in the first 2 years of life, the central regions of the WM are already visible by DTI at birth. Many authors [23–25] have described WM anisotropy and mean diffusivity throughout the development process as a reference with which to characterize the early stages of maturation including the premyelinating state.

Partridge et al. [22] serially examined WM development by DTI in 14 premature infants with no evidence of WM abnormalities by conventional MRI. More significant age-related changes in DTI values were identified in the transverse fiber tracks of the corpus callosum than in other WM pathways. Xueying et al. [26] compared WM maturation patterns in major fiber pathways between 60 preterm infants and 25 term controls with normal MRI and neurologic examinations at term-equivalent age using diffusion parameters, FA, and apparent diffusion coefficients. They showed that the increased FA in the preterm infants at term-equivalent age was significantly different from the decreased FA in the term infants, suggesting that prematurity is an independent factor of accelerated maturation of WM in the extrauterine environment compared to term controls.

However, there have been few DTI studies about the predictive value of abnormal WM lesions prior to the manifestation of hemiparesis in preterm infants with high risk factors. Fundamental questions remain to be addressed to predict long-term developmental outcomes at term-equivalent age, limiting our ability to assess the therapeutic interventions needed during critical periods of development. DTI might add another piece to the puzzle of pathophysiology preceding developmental delay in high-risk preterm infants with WM abnormalities associated with prematurity. The early identification of candidates at risk of developing CP or abnormal WM maturation is helpful in selecting infants for potential therapeutic interventions in order to improve long-term outcomes. In the present study, infants having WM abnormalities in the presence of CP showed a decrease in the FA of diffusion tensor values at term-corrected age, particularly in the regions of the centrum semiovale, the posterior limb of the internal capsule, and the corpus callosum.

Our findings are similar to the results of earlier studies. Murakami et al. [27] examined DTI with fiber tracking for corticospinal tracts in 10 patients with WM abnormalities associated with prematurity during infancy to predict clinical motor functions at the early stage of development as a biomarker. Disturbance to the posterior limb of the internal capsule is especially known to increase vulnerability to hypoxic ischemic injury in infants. De Bruïne et al. [28] confirmed a strong correlation between those low FA values of the posterior limb of the internal capsule at term-equivalent age and subsequent psychomotor delay at the age of 2 years in very preterm infants. Roze et al. [29] determined the association between later development of spastic CP and early perturbation of DTI values. They showed that asymmetries in FA within 4 weeks after birth were predictive of unilateral spastic CP in preterm infants with periventricular hemorrhagic infarction. Rose et al. [30] examined the WM microstructures of six subcortical regions on DTI in 66 VLBW preterm infants at near-term age. They found a relationship between lower mean diffusivity of the thalamus and higher total bilirubin, which is known to be a risk factor of adverse neurodevelopment. Son et al. [31] revealed corticospinal tract disruption prior to clinical manifestations of hemiparetic CP, even though the conventional brain MRI of patients showed no abnormalities. In addition to being a predictor of motor outcomes, several studies suggest a relationship between WM microstructure at term-equivalent age and cognitive outcomes in children and adolescents born very preterm [32, 33]. Perinatal brain damage of WM abnormalities associated with prematurity may impact the normal maturation of cortical grey matter, which reflects the disorganized and disrupted axons. Woodward et al. [14] stressed the importance of cerebral WM connectivity for later neurocognition such as intelligence, language, and executive function. Consistent with data above, our study showed that four of five infants in the PVL with CP group had the expected drop on the Bayley-III cognitive and language scores at 2 years of age, preceded with low FA values in WM t term age. Skranes et al. [6] investigated the relationships between low scores on the Wechsler Intelligence Scale for Children-III test and low FA values in several WM areas in the VLBW group. Although FA analysis of our DTI study was not properly differentiated to the cognitive assessment, early perturbation of DTI values may be associated to later cognitive development in different brain areas.

## Conclusions

This study demonstrates low FA values of ROIs in DTI are related with later development of spastic CP in preterm infants with WM abnormalities. A quantitative approach using DTI in specific WM might provide prognostic values for the brain development in preterm infants. The value of DTI in predicting long-term infant neurodevelopmental outcomes should be analyzed in a larger cohort.

## Additional file

Additional file 1: Figure S1. The representative axial images on T2 flair image (A) and tractography of motor fibers on DTI (B) are shown in preterm infants with white matter abnormalities without cerebral palsy. (PPTX 563 kb)
