# Supplementary figures and images for: Neonatal diffusion tensor brain imaging predicts later motor outcome in preterm neonates with white matter abnormalities
Source: Ital J Pediatr. 2016 Dec 1;42:104. doi: 10.1186/s13052-016-0309-9 (PMC5134238; doi:10.1186/s13052-016-0309-9)

## Slide 1
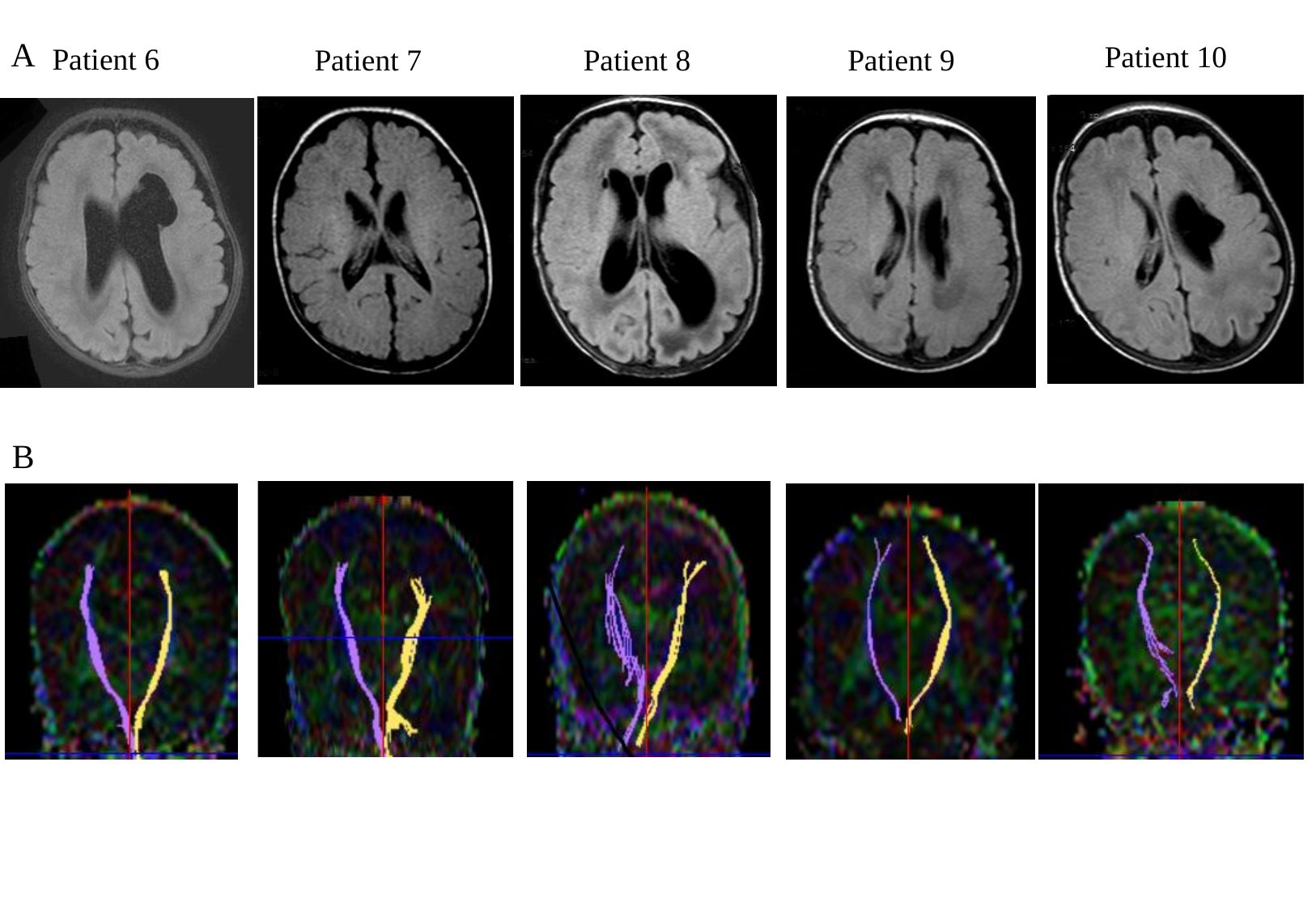

1
A
Patient 10
Patient 6
Patient 7
Patient 8
Patient 9
B

Supplement: Additional file 1: Figure S1. — The representative axial images on T2 flair image (A) and tractography of motor fibers on DTI (B) are shown in preterm infants with white matter abnormalities without cerebral palsy. (PPTX 563 kb) [file 13052_2016_309_MOESM1_ESM.pptx]
